# Supplementary material for: A Marine Alkaloid, Ascomylactam A, Suppresses Lung Tumorigenesis via Inducing Cell Cycle G1/S Arrest through ROS/Akt/Rb Pathway
Source: Mar Drugs. 2020 Sep 27;18(10):494. doi: 10.3390/md18100494 (PMC7599880; doi:10.3390/md18100494)
Supplement: Supplementary file 1 [file marinedrugs-18-00494-s001.pdf]

# Supplementary Materials

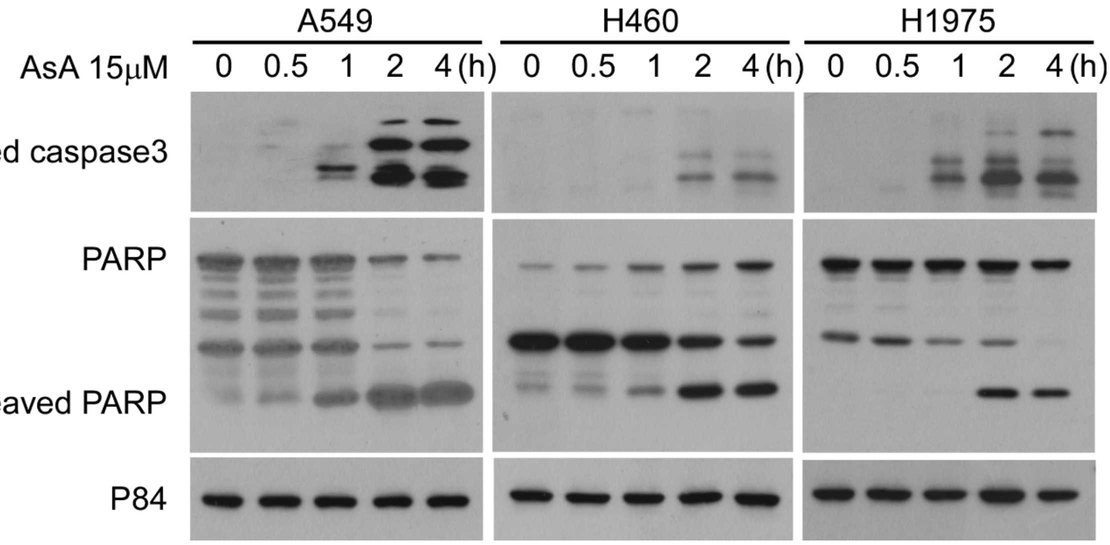


**Figure S1.** Effects of AsA on apoptotic markers in NSCLC cells. Western blotting analysis of caspases3 and PARP in A549, NCI-H460 and NCI-H1975 cells were treated with AsA (15 μM) for 0, 0.5, 1, 2 and 4 h.
